# Supplementary material for: Multi‐Scale Volumetric Dynamic Optoacoustic and Laser Ultrasound (OPLUS) Imaging Enabled by Semi‐Transparent Optical Guidance
Source: Adv Sci (Weinh). 2023 Dec 20;11(9):2306087. doi: 10.1002/advs.202306087 (PMC10953719; doi:10.1002/advs.202306087)
Supplement: Supplementary file 1 — Supporting Information [file ADVS-11-2306087-s005.pdf]

ADVANCED SCIENCE
Open Access

Supporting Information

for *Adv. Sci.*, DOI 10.1002/advs.202306087

Multi-Scale Volumetric Dynamic Optoacoustic and Laser Ultrasound (OPLUS) Imaging Enabled by Semi-Transparent Optical Guidance

Daniil Nozdriukhin, Sandeep Kumar Kalva, Cagla Özsoy, Michael Reiss, Weiye Li, Daniel Razansky and Xosé Luís Deán-Ben*

Supporting Information

Multi-scale volumetric dynamic optoacoustic and laser ultrasound (OPLUS) imaging enabled by semi-transparent optical guidance

Daniil Nozdriukhin[1,2], Sandeep Kumar Kalva[1,2], Cagla Özsoy[1,2], Michael Reiss[1,2], Weiye Li[1,2], Daniel Razansky[1,2], Xosé Luís Deán-Ben[1,2]

[1]Institute of Pharmacology and Toxicology and Institute for Biomedical Engineering, Faculty of Medicine, University of Zurich, Switzerland

[2]Institute for Biomedical Engineering, Department of Information Technology and Electrical Engineering, ETH Zurich, Switzerland

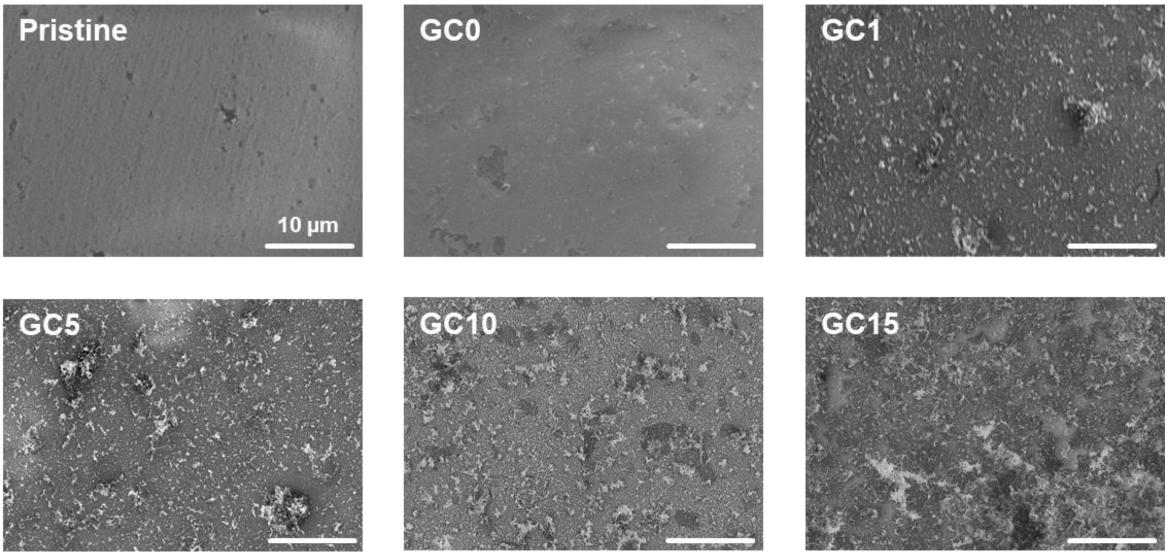

Supplementary Figure 1. SEM images of the fiber tip surface. GC0 - polyelectrolyte pillow, GC# - Gold-Carbon coating, # - amount of gold-carbon bilayers.

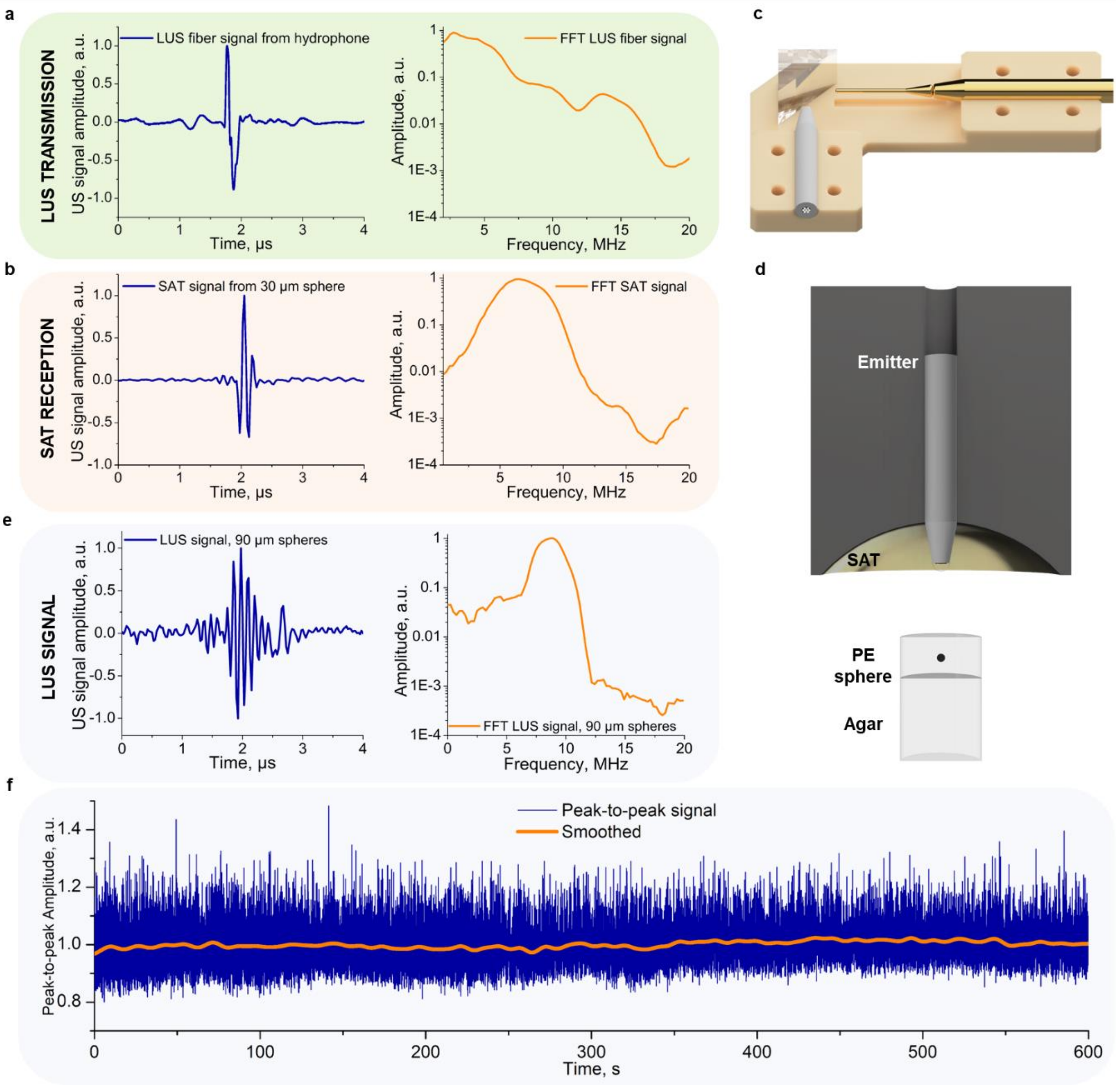

Supplementary Figure 2. Transmission and reception bandwidth. (a) LUS signal from the GC15 fiber acquired with a hydrophone. The time domain signal (left) and Fourier transform of this (right) are shown. (b) OA signal from a single sub-resolution (30 µm) sphere acquired with an element of the 512-element spherical array transducer (SAT). The time domain signal (left) and Fourier transform of this (right) are shown. (c) The layout of the experimental setup that used to measure the emission bandwidth and dependence of the signal on a number of gold-carbon bilayers. (d) Layout of the experimental setup to measure the bandwidth of the transducer. (e) US signal band of 90 µm spheres in time and frequency domain acquired with the spherical array transducer with semitransparent emitter. (f) A mean peak-to-peak amplitude of the ultrasound signal over the elements of 512-element spherical array transducer emitted by the semitransparent transducer. The input energy is 15 mJ/pulse at 800 nm illumination at a 100Hz repetition rate. The signal shows good photostability.

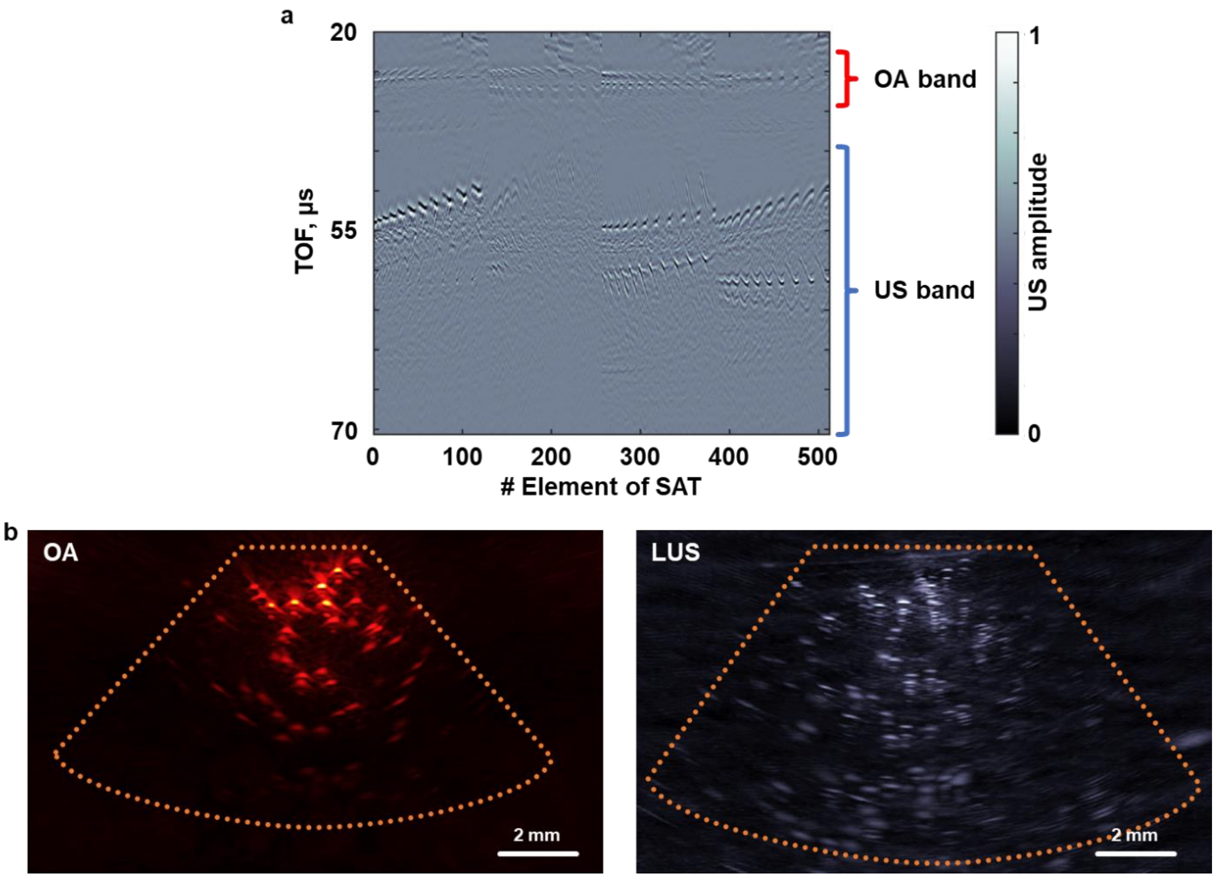

Supplementary Figure 3. (a) Raw time-domain signals (sinogram) acquired with the spherical array, the x-axis represents a number of the receiving element and the y-axis corresponds to the time of flight. The OA signal arrives first after approx. 27 µs delay from the laser trigger pulse, and the US echo arrives at ca. 55 µs delay and lasts longer. (b) Typical lateral broadening of the light (OA) and laser ultrasound (LUS) beams in the tissue-mimicking phantom consisting of 1% agar gel with 1.3% of intralipid with 90 µm black polyethylene spheres embedded.

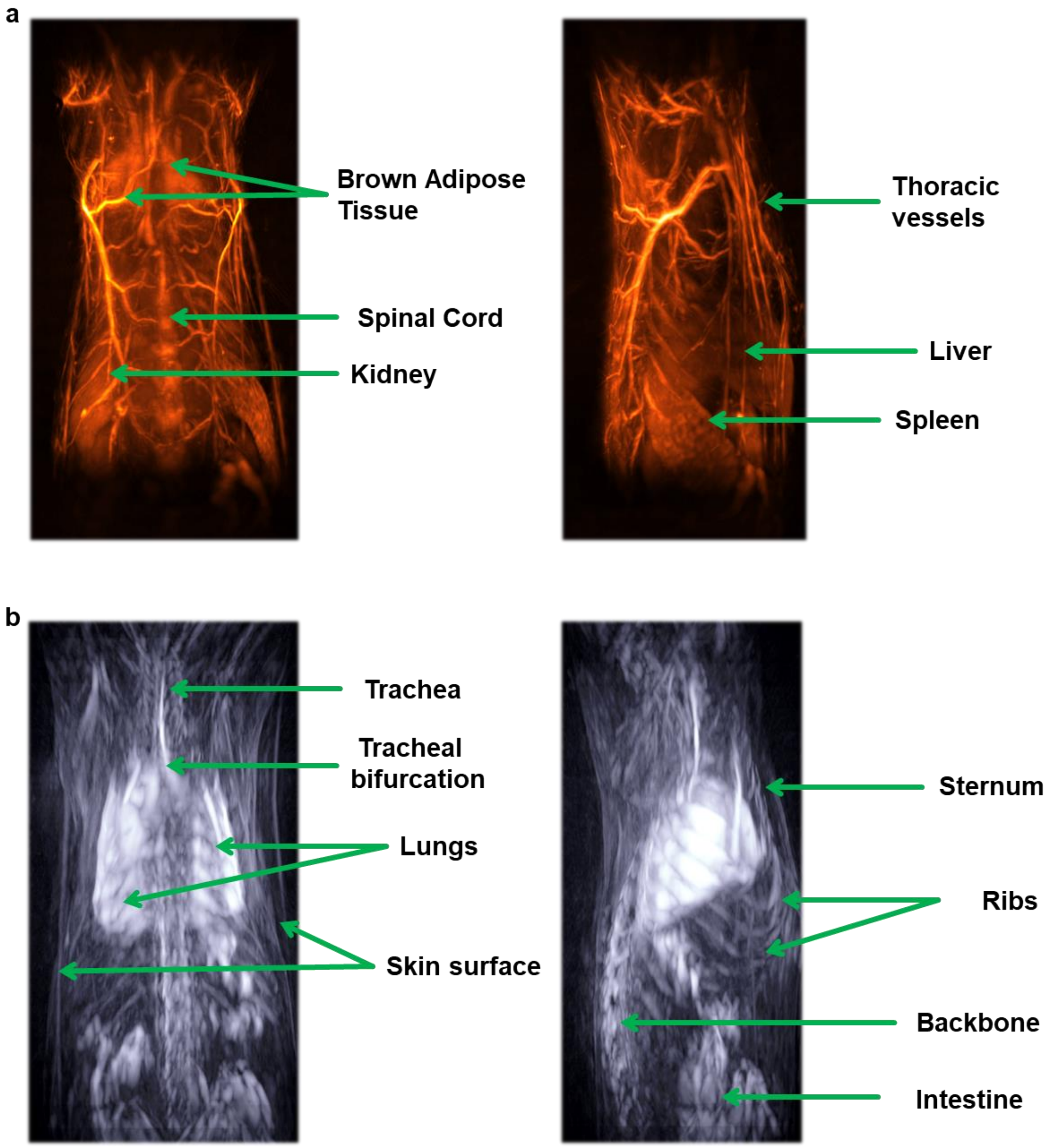

Supplementary Figure 4. (a) OA maximum intensity projection (MIP) of the mouse in 2 projections with visible structures labelled. (b) LUS MIP of the mouse in 2 projections with visible structures labelled.

Supplementary Equation 1:

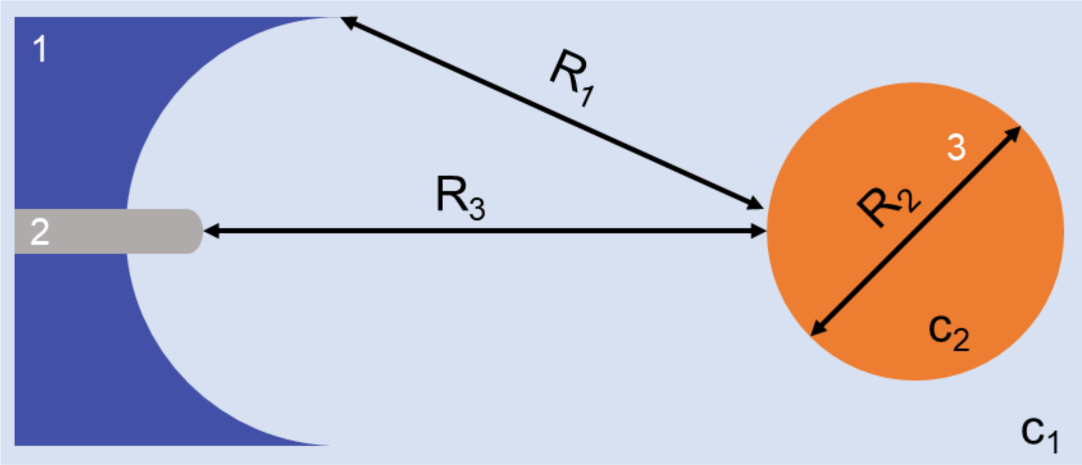

Let's assume that the spherical array transducer (1), semitransparent LUS emitter (2) and the body of interest (3) are placed in the medium with the speed of sound c_1, while the speed of sound inside the body is c_2. The distance from the transducer to the body is R_1, the size of the body is R_2 and the distance from the emitter to the body is R_3. Let's also assume that the optoacoustic excitation is an instant process since the speed of light is much higher than the speed of sound in medium. Let's also simplify that the speed of sound in the body is constant (average of all the sound velocities in the body, isotropic), the speed of sound in the medium is also constant and the light penetration depth could cover the entire body of interest (which gives us an upper limit to the non-overlapping conditions).

To prevent the overlap of the optoacoustic and ultrasound signal, the last optoacoustic signal (from the distance of $R_1 + R_2$) should arrive earlier than the first ultrasound signal (from the distance R_1).

Considering all the conditions, the time of flight for OA and US signals will be:

$$t_{OA} = \frac{R_2}{c_2} + \frac{R_1}{c_1} + \boxed{\frac{R_3 + R_2}{c_{light}}} = \frac{R_2}{c_2} + \frac{R_1}{c_1}$$

and

$$t_{LUS} = \frac{R_1}{c_1} + \frac{R_3}{c_1}$$

Hence,

$$t_{OA} < t_{LUS} \Leftrightarrow \frac{R_2}{c_2} + \frac{R_1}{c_1} < \frac{R_1}{c_1} + \frac{R_3}{c_1} \Leftrightarrow R_3 > \frac{c_1}{c_2} R_2$$

It means that the distance from the focus of the transducer to the emitter position should be larger than the body attempted to image or the desired penetration depth since the relation of the speed of sound in water to the speed of sound in soft tissue ~1.

In the experimental conditions of the manuscript, a finger, a mouse and a palm were imaged with the emitter positioned at 30 mm from the focus, which satisfies the condition.

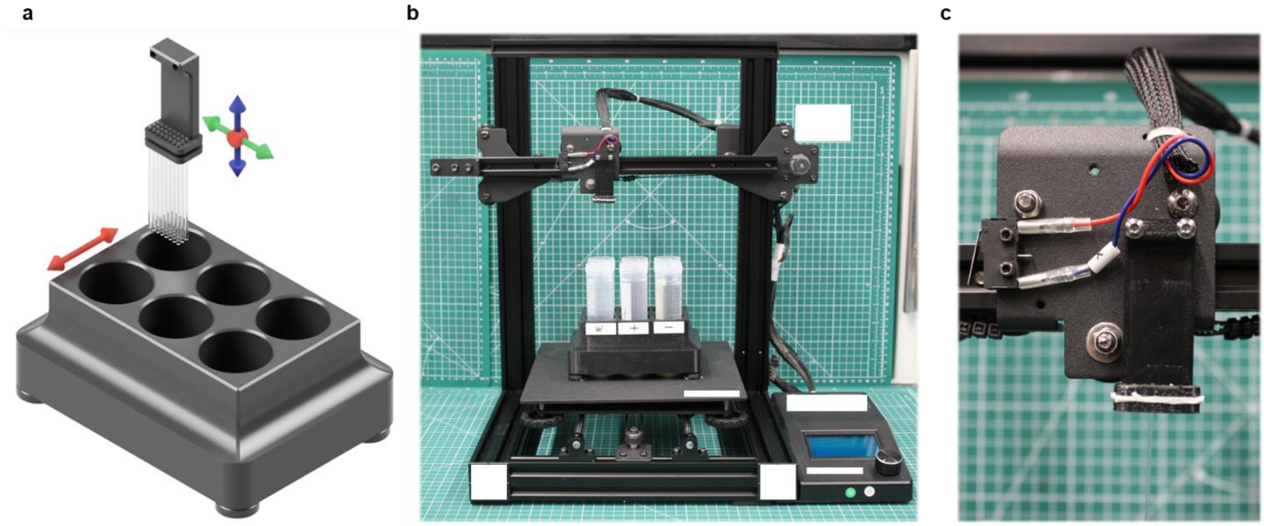

Supplementary Figure 5. (a) Layout of a dip-coater with multiple wells. The fiber holder with the ability to carry 24 fiber pieces can move in X-Z directions while the tube rack with 6 slots can move in Y direction. (b) Photograph of a modified 3D printer for dip-coating purposes. (c) Close-up view of the fiber holder mounted instead of the standard filament extruder.

Code Example:

```
;STARTING SEQUENCE
G0 Z50; RISE THE FIBER HOLDER
M107;TURN OFF THE FAN
G28 ; HOME ALL AXIS
G0 F700 Z200; REACH THE HEIGHT
G0 F700 Y200; PULL THE PRINTING TABLE TO LOAD THE TUBE RACK
M117 LOAD THE SAMPLE AND CLICK THE KNOB
M0; PAUSE TILL CLICK
;END OF STARTING SEQUENCE

;DIPPING SEQUENCE
G0 F1800 X74 Y106; TRAVEL TO A VESSEL WITH XY COORDINATES
M117 DIPPING
G0 F1000 Z122; DIP INTO 2-1 VESSEL
G4 S600; WAIT FOR 600s IN 2-1 VESSEL
G0 F1000 Z200; RISE THE FIBER HOLDER
;END OF DIPPING SEQUENCE

;FINISHING SEQUENCE
G0 Z200; RISE THE FIBER HOLDER
G0 Y200; MOVE THE TUBE RACK TO THE FRONT
M84; TURN OFF THE MOTORS
M0; PAUSE TILL CLICK
```

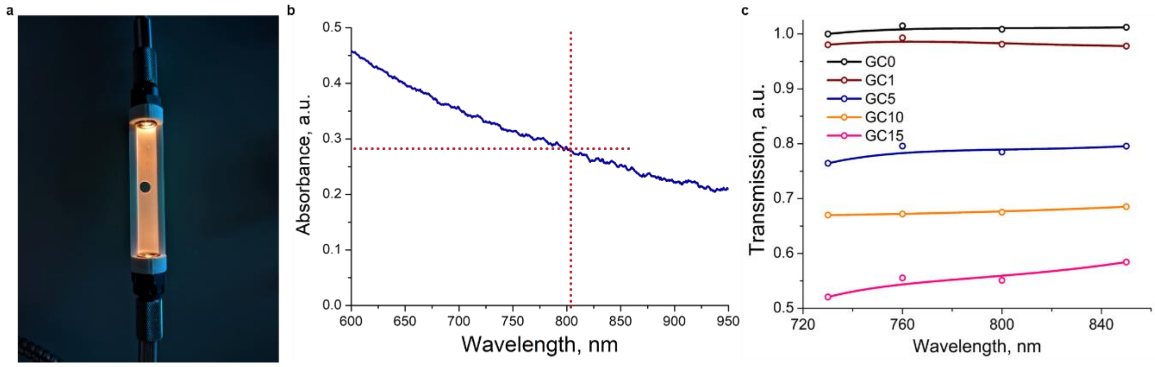

Supplementary Figure 6. (a) OPLUS fiber bundle cuvette connected to a portable fiber optic spectrometer with a halogen lamp as an excitation source. (b) NIR spectrum of the GC15 OPLUS fiber bundle. The absorbance of 0.28 at 800 nm corresponds to 52.5% of light transmission. (c) The dependance of light transmission on the number of layers for different wavelengths.
